# The efficacy of psychodynamic psychotherapy for young adults: a systematic review and meta-analysis

**DOI:** 10.3389/fpsyg.2024.1366032

**Published:** 2024-09-04

**Authors:** Antonella Trotta, Andrew J. Gerber, Felicitas Rost, Sarah Robertson, Avi Shmueli, Rosine J. Perelberg

**Affiliations:** ^1^School of Health and Social Care, University of Essex, Colchester, United Kingdom; ^2^Social, Genetic & Developmental Psychiatry Centre, Institute of Psychiatry, Psychology & Neuroscience, King’s College London, London, United Kingdom; ^3^British Psychoanalytical Society, London, United Kingdom; ^4^Columbia University College of Physicians & Surgeons, New York, NY, United States; ^5^Silver Hill Hospital, New Canaan, CT, United States; ^6^School of Psychology and Psychotherapy, Faculty of Arts and Social Sciences, The Open University, Milton Keynes, United Kingdom; ^7^Psychoanalysis Unit, Research Department of Clinical, Educational and Health Psychology, University College, London, United Kingdom

**Keywords:** efficacy, meta-analysis, outcome studies, psychoanalytic psychotherapy, systematic review

## Abstract

**Objective:**

One in six young adults presents with at least one mental health problem. However, so far, little attention has been directed to the mental health needs and the efficacy of therapeutic interventions for young adults. We conducted a systematic review and meta-analysis of the type, quality and efficacy of psychoanalytic psychotherapy for young people.

**Method:**

We searched the PsycInfo, PubMed, Embase, and Cochrane databases to identify all the published randomized controlled trials (RCT), and naturalistic and observational studies of psychodynamic or psychoanalytic psychotherapies. We calculated the standardized mean difference in scores of psychodynamic interventions versus control conditions, adopting a random effects model (Hedges’ *g*).

**Results:**

We identified 22 eligible studies, including 14 RCTs, and 8 naturalistic studies. Statistical analyses showed no significant difference between psychodynamic psychotherapy and other comparison treatments (psychotherapy or pharmacological interventions) for young adults (Hedges’*g* − 0.34 [95% CI: −0.991;-0.309], *p* = 0.304). Nevertheless, there was a significant effect of psychodynamic psychotherapy when compared with control conditions (waiting list or treatment as usual) for target symptoms (Hedges’*g* − 1.24 [95% CI: −1.97;-0.51], *p* < 0.001).

**Conclusion:**

Our systematic review highlights important clinical implications in identifying the efficacy of psychoanalytic interventions for specific at-risk groups and suggests developing prevention strategies for mental health problems in young adulthood across cultures and context.

## 1 Introduction

One in six young adults suffer from at least one mental health problem (Patel et al., 2007), with rates of anxiety and depression being the highest in 17- to 19-year-olds (Sadler et al., 2018). Furthermore, the pandemic has increased health inequalities and challenges for young people worldwide. Young people’s mental health has been particularly affected through the impact on educational, working, social, and family daily life (e.g., Banks and Xu, 2020; McGinty et al., 2020; Sampogna et al., 2021; Silva Junior et al., 2020).

Young adulthood, spanning 18–27 years-of-age, is a transition period, wherein the young individual moves from adolescence into adulthood and forms their position and identity within adult society. These age boundaries are arbitrary markers of both the developmental and social processes encountered during this phase of life (Arnett, 2011). It is a time of consolidation of intellectual and emotional capacities for the young person in order to meet the demands of life and society, as well as a time when they must accomplish internal and external tasks for the self and others (Perelber, 1993).

Encounters with rapid psychobiological changes and questions related to the development of identity often increases the levels of anxieties in this population (Schulenberg et al., 2004). It is not surprising that young adults present higher prevalence of mental health problems than children and adults and in turn, these might lead to adverse socio-economic consequences in later life (Patel et al., 2007). In fact, 75% of adults with a mental disorder report an age of onset younger than 24 years (Kessler et al., 2005).

Nonetheless, for many years young adults have been a neglected population within research into therapy outcomes (Lindgren et al., 2010; Philips et al., 2006). This might be explained by the widespread difficulties for young people in accessing care (Hagell et al., 2018) as well as by the methodological challenges of researching the therapeutic process without impacting the procedure under observation (Leuzinger-Bohleber and Kachele, 2015). Given the increasing demand for mental health services for young adults, there is an urgent need for effective interventions for this population.

Previous meta-analyses have focused on the effect of evidence based treatments for specific mental health problems in youth, including anxiety, depression, attention-deficit hyperactivity disorder (ADHD), and conduct disorders (Cuijpers et al., 2009, 2020; Weisz et al., 2019) and highlighted the importance of examining both the main effects of psychotherapy as well as specific treatment approaches.

Increasing research evidence suggests that psychoanalytic psychotherapy is an effective treatment for people with psychological difficulties, showing significant improvements both in clinical symptoms and overall functioning at the end of treatment (e.g., Nemirovski Edlund and Carlberg, 2016; Steinert et al., 2017). However, the majority of these studies have been conducted in children (Abbass et al., 2013; Midgley et al., 2021) or adult populations (de Maat et al., 2008; Leichsenring and Leibing, 2003).

Although a number of manualized and short-term treatments are considered as psychodynamic (Leichsenring et al., 2015; Seybert et al., 2011; Yakeley, 2014), in clinical practice, the majority of psychodynamic psychotherapies do not follow a structured protocol and, due to the specificity of the intervention, have received limited supporting evidence from randomized controlled trials (RCTs) compared to other therapeutic modalities.

Despite RCTs being considered the ‘gold standard’ to assess the efficacy of psychological therapies, researchers have argued that evidence beyond that provided by RCTs is required to empirically support psychotherapy treatments, shifting the focus from whether an intervention works to ‘what works for whom?’ (Fonagy et al., 2014; Fonagy, 2015).

To date, no systematic review and meta-analyses have summarized the available literature on the efficacy of psychoanalytic therapy for young adults and, therefore, it is not known whether psychodynamic psychotherapies are effective for this population.

The aim of our study was therefore to address that paucity. The main objectives were to: (i) conduct a systematic review and appraisal of the current research evidence; (ii) conduct a meta-analysis to compare the effect of psychoanalytic therapy to treatments with established efficacy for this specific population and to control conditions using reliable and valid outcome measures.

Our review was guided by three specific review questions: (i) What are the key outcome measures in psychoanalytic therapies? (ii) How are the outcome of psychodynamic therapies for young people evaluated? (iii) What is the empirical evidence for psychoanalytic interventions for young adults?

## 2 Methodology

### 2.1 Search terms and inclusion criteria

The electronic databases Ovid, Embase, PubMed, Psych INFO and Cochrane were used to identify all published studies of “psychodynamic” or “psychoanalytic” psychotherapies. These include a family of psychotherapeutic approaches focused on the understanding of personal and relational patterns, the potential link between the person’s past and present experiences, the expression of feelings, and the exploration of defense patterns (Gabbard, 2010; Leichsenring et al., 2023).

The electronic search initially includes papers indexed by the aforementioned web-based databases as of January 2020 and was updated in July 2023. In order to obtaine a comprehensive list, we also manually screened relevant textbooks, systematic reviews, meta-analyses and reference checklists of studies included and consulted experts in the field. Supplementary material contains the full search strings. The search strategy included terms referring to young adult population (e.g., ‘young’, ‘adult’ and ‘youth’), mental health problems (e.g., psychiat* or mental* or psychol* or diagnosis) and psychoanalytic treatments.

We adopted the Preferred Reporting Items for Systematic Reviews and Meta-Analyses guidelines (PRISMA; Page et al., 2021). Because of the lack of research on outcomes of psychotherapy for young adults generally, we included RCTs, quasi-experimental studies, and naturalistic evaluations whose quantitative measurement of therapy process or outcomes were reported at ‘baseline’ (T1) and at a later time-point (T2).

We did not adopt a sample size cut-off in the selection of the studies in order to capture a broader range of quality and reflect the heterogeneity that occurs in clinical practice (Turner et al., 2013). Inclusion criteria were: studies of psychodynamic or psychoanalytic psychotherapies with published articles in English and in peer reviewed journals, subjects were between 18 and 27 years-old at the start of therapy and receiving treatment for mental health issues, to capture the extended psychological challenges of adolescence and young-adulthood (Giedd et al., 1999). We excluded studies of young populations suffering from organic mental disorders, and where no quantitative measurement of therapy outcomes were provided.

We defined intervention as therapy that was psychoanalytic or psychodynamic (Gabbard, 2010). We included short-term psychodynamic therapy, typically between 12 and 24 weekly sessions as well as longer term psychoanalytic treatments, up to 8 years duration. Comparators were any form of psychotherapy or pharmacotherapy that has proven its efficacy against the corresponding mental disorders on the basis of published standards and guidelines (Chambless and Hollon, 1998; Nathan and Gorman, 2015). If the same study had published more than one article we included the publication of the main outcome instead of the follow-up, or secondary analyses.

Given the heterogeneity of design, populations and measures of the studies included, we synthesized the findings in a narrative form, and grouped the outcomes measures into five main thematic categories across the studies included (clinical symptoms, recovery/relapse, psychosocial functioning, personality structure and interpersonal relationships).

### 2.2 Assessment of study quality

We applied the Randomized Controlled Trial of Psychotherapy Quality Rating Scale (RCT-PQRS; Kocsis et al., 2010), to rate the methodological quality of RCTs of psychotherapy. The RCT-PQRS was developed by an expert committee with different allegiances (e.g., CBT, psychodynamic therapy, pharmacology) and based on pre-existing quality measures of RCTs (Thoma et al., 2012). The scale was designed to evaluate the quality of psychotherapy trials and has shown good psychometric properties in terms of internal consistency and external validity (Jadad et al., 1996; Moher et al., 2001; Moncrieff et al., 2001). It includes 24 items each assessing a specific element of the study design and methodology, with scores ranging between 0 and 2, yielding a maximum score of 48. For the purpose of the study, we used the total score of the 24 items as the primary quality measure. A quality score of 24 or above is considered to represent a cut-off for a “reasonably well-done study” (Gerber et al., 2011).

For naturalistic studies, we used a quality assessment tool designed by the National Institute for Health Research (National Institutes of Health, 2014), which allows a critical appraisal and assessment of the internal validity of each study, whether the study findings can be attributed to the intervention. The tool includes a total of 14 questions to assess selection, information, measurement, and confounding bias (for example differences patients’ characteristics at the start of the intervention). Each study was rated as 1 (Yes) or 0 (No) on each question, based on whether the item criteria was met or not. A total score was calculated summing the single questions and each study was classified as ‘poor’ (total score below 5), ‘fair’ (between 6 and 10) or ‘good’ quality (total score above 10). A low score of the study quality corresponds to greater risk of bias and, viceversa, higher quality rating indicates a lower risk of bias.

### 2.3 Statistical analysis

We used the following inclusion criteria: RCTs of psychoanalytic therapy for young adults, comparing psychoanalytic therapy to another treatment with established efficacy or control conditions, and using quantitative outcome measures. The primary outcomes were “target symptoms,” which included various measures for the mental disorders being studied, such as anxiety symptoms in anxiety disorders or obsessive-compulsive behavior measures in obsessive-compulsive disorder. The secondary outcomes included psychosocial functioning (such as personality, social, and occupational functions).

We conducted two separate meta-analyses: (i) first, we compared the effect of psychodynamic psychotherapy to other treatments on outcomes, (ii) second, we compared the effect of psychodynamic therapy to control conditions (e.g., waitlist, treatment as usual) for chosen primary and secondary outcomes.

Data extracted were: study authors and year of publication; sample characteristics (size, psychiatric diagnosis, diagnostic measures); details of interventions (including length of treatment) and control group condition; primary and secondary outcome measures; follow-up duration, and effect sizes. In case of missing data, we contacted the corresponding authors.

Statistical analyses were carried out using Stata (Version 15; StataCorp, 2017). Due to between-study heterogeneity, we adopted a random effects model, which is recommended for meta-analysis of psychological therapies (Higgins and Green, 2008).

For continuous outcomes, pooled standardized mean difference (SMD) was calculated with 95% confidence intervals. SMD was utilized in order to pool together means across studies utilizing different outcome measures and was calculated by dividing the mean difference in outcome between participants allocated to psychodynamic psychotherapy and those allocated to the comparison intervention or control group by the pooled within-groups standard deviation. If the outcomes were expressed as an event proportion, they were converted to odds ratios and then subsequently converted to Hedges’ *g* (Hedges, 1981).

Cohen’s criteria for the interpretation of effects was used: 0.2 suggests a small effect, 0.5 a medium effect, and 0.8 for a large effect (Higgins and Green, 2008). The results are displayed using a forest plot.

Heterogeneity between studies was assessed with the Cochran’s *Q* test (DerSimonian and Laird, 1986). A statistically significant Q value indicates true heterogeneity in effect sizes beyond random error; the *I^2^* statistic was calculated to express the proportion of variation among studies that was due to heterogeneity (Higgins et al., 2003). Heterogeneity was categorized as low (0–40%), moderate (30–60%), substantial (50–90%), or considerable (75–100%; Deeks et al., 2019).

To further explore heterogeneity, we carried out meta-regression analyses to test effects of potential moderator variables such as: year of publication, quality score (total score of the RCT-PQRS), age, sex, outcome measure (self-report vs. interview), recruitment methodology (community compared with clinical populations compared with mixed samples), intent-to-treat compared with completer analyses, diagnosis, patient-per therapist ratio (to investigate the presence of bias from therapist effects), and average sample size per group to assess the small study bias (Nüesch et al., 2010). All moderators were otherwise entered separately to the meta-regression analysis due to the small number of included studies (Borenstein et al., 2010).

We conducted sensitivity analyses to examine the effect of outliers, defined as those studies displaying a 95% CI that did not overlap with the 95% CI of the pooled effect size. Publication bias was assessed by visual inspections of funnel plots. In addition, Egger’s test of publication bias was used to investigate whether there was a tendency for selective publication of studies (Egger et al., 1997).

A significance level of *p =* 0.05 was used for the random-effects model, homogeneity, publication bias, and meta-regression analyses.

## 3 Results

### 3.1 Selection of studies

**Figure 1:**
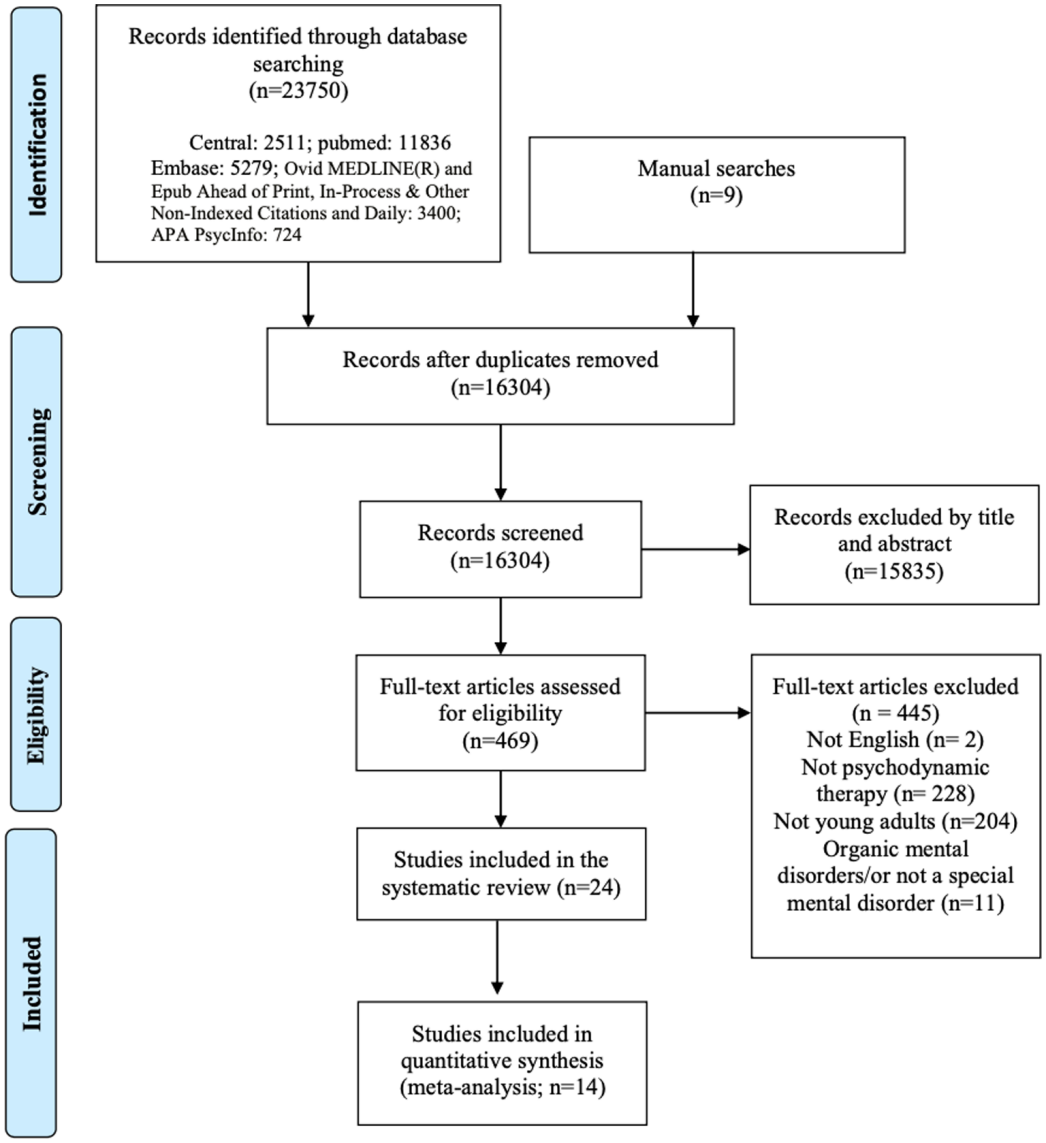
Flow chart describing the selection process.

The PRISMA flowchart showing the inclusion process is illustrated in Figure 1. A total of 23,759 related documents were initially retrieved. After reading the titles and abstracts, 469 studies remained to be read in full. According to the inclusion and exclusion criteria, the results were filtered in order to select the eligible studies. A total of 22 published papers from the database searches fulfilled the selection criteria. Of those, 14 studies were RCTs, and eight studies were naturalistic studies.

### 3.2 Characteristics of studies included

Data on a total of 2,649 young adults were included in the studies (2,063 in the treatment and 586 in the control conditions). A summary of the main characteristics of the studies included can be found in Table 1. Ten studies compared psychodynamic therapy with another psychotherapy treatment, including CBT, Counseling, Cognitive-Analytic Therapy (CAT), Family Therapy (FT), Dialectical behavior therapy (DBT). One study compared psychodynamic therapy with pharmacological intervention.

**Table 1:** Characteristics of studies included in the systematic review.

Study	Baseline characteristics			Intervention			Outcome measure	Area of outcome
	Age (SD)	Sex (Female/Male)	Diagnosis	Treatment and control condition	Number of patients	Sessions		
**Randomized controlled trial (RCT)**								
Ajilchi et al. (2016) (Iran)	19-40 (84.4%19-29)	9/23	Major depressive disorder (MDD)	Intensive short-term dynamic psychotherapy (ISTDP)	16	15	Beck depression inventory (BDI-II; Beck et al., 1996)	Primary outcome: depression
				Wait-list	16		Wisconsin Card Sorting Test (WCST; Heaton et al., 1993) Stroop Task (MacLeod, 1991)	Secondary outcome: executive functioning
Bachar et al. (1999) (Israel)	24.1 (3.3)	33/0	Bulimia Nervosa (BN) and Anorexia (AN)	Self- psychological treatment (SPT)	14	48	DSM symptomatology scale for anorexia and bulimia (DSM SS), EAT 26 (Eating attitudes test; Garner et al., 1982)	Primary outcome: general psychiatric symptoms
				Cognitive orientation treatment (COT)	12	48	BSI (Brief symptom inventory; Derogatis and Spencer, 1982)	
				Control/nutritional counseling only (C/NC)	7	12	Selves questionnaire (Higgins, 1987)	
							Symptom Checklist-90-R (SCL-90-R; Derogatis and Unger, 2010)	Secondary outcome:symptomatic remission
Cooper et al. (2003) (United Kingdom)	27.69 (5.36)	193/0	Post-partum depression	Counseling	193	18	Edinburgh Postnatal Depression Scale, (EPDS; Cox et al., 1987); Structured Clinical Interview for DSM-III-R (SCID; Spitzer et al., 1992); Therapist Rating Scale (Silove et al., 1990)	Primary outcome: maternal mood
				Cognitive behavioral therapy (CBT)				
				Psychodynamic psychotherapy				Secondary outcomes: depression therapist adherence
				Control				
Dare et al. (2001) (Denmark)	26.3 (6.7)	82/2	Anorexia nervosa (AN)	Focal psychodynamic psychotherapy (FPP)	12	24.9 (13.0)	Morgan-Russell psychiatric interview (Morgan and Hayward, 1988).	Primary outcome: weight gain
				Cognitive-analytic therapy (CAT)	13	12.9 (7.0)	Body mass index (BMI)	
				Family therapy (FT)	16	13 (8.6)		
				Low contact, `routine' treatment (LRT)	13	10.9 (0.5)		
Doering et al. (2010) (Austria, Germany)	27.46 (6.8)	104	Borderline personality disorder (BPD)	Transference-focused psychotherapy (TFP)	104	48.5	German version of the Cornell Interview for Suicidal and Self-harming behavior (CISSB; Clarkin, 1998a); SCID-I and -II (American Psychiatric Association, 1994)	Primary outcomes: Number of participants who dropped out; Suicide attempts
							Global Assessment of Functioning Scale (GAF; American Psychiatric Association, 1994) BDI; State–Trait Anxiety Inventory (STAI, Spielberger et al., 1999); BSI; Cornell Revised Treatment History Inventory (CRTHI; Clarkin, 1998b)	Secondary outcomes: DSM–IV diagnostic criteria for borderline personality disorder and number of comorbid Axis I and II diagnoses Psychosocial functioning General psychopathology Self-harming behavior Psychiatric in-patient admissions.
Garner et al. (1993) (Canada)	24.15 (4.19)	50/0	Bulimia nervosa (BN)	Supportive-expressive therapy (SET)	50	19	Eating attitude test; Eating disorder examination (Cooper and Fairburn, 1987);	Primary outcomes: Frequency of vomiting; Binge eating episodes
				CBT			Eating disorder inventory (Garner, 1991); SCL-90-R; Borderline syndrome index (Conte et al. 1980); Rosenberg-self esteem scale (Rosenberg, 1965; BDI; Millon Clinical Multiaxial Inventory) (Millon, 1982); Social adjustment scale-self-report (Weissman et al., 1978).	
Moghadam et al. (2015) (Iran)		0/38	Social phobia	Short-term dynamic psychotherapy	13	25	Social Phobia Inventory (Connor et al., 2000; SPIN)	Primary outcome: Social phobia
				Sertraline	11	12 weeks	Global Clinical Impression-Severity and Improvement (Zaider et al., 2003; CGI-S, CGI-I)	Secondary outcomes: General psychopathology
				Waiting list	14		GAF Scale	
Mowlaie et al. (2018) (Iran)	20.93 (1.50)	30/0	Adult separation anxiety disorder (ASAD)	Brief empathic psychotherapy (BEP)	30	12	Adult Separation Anxiety Questionnaire (ASA-27; Manicavasagar et al., 2003)	Primary outcomes: Anxiety Depression
				Affect phobia therapy (APT)			Depression, anxiety, and stress scale-21 (DASS-21; Lovibond and Lovibond, 1995)	
							Structured clinical interview for separation anxiety symptoms (SCI-SAS; Cyranowski et al., 2002)	
							GAF Scale	
Orvati Aziz et al. (2020) Iran	24.92 (5.25)	91.7% female in the integrative therapy group	Generalized anxiety disorder	Integrative therapy (short-term psychodynamic psychotherapy and cognitive-behavioral therapy) cognitive-behavioral therapy	36	15	Hamilton rating scale for anxiety (HRSA; Hamilton, 1959)	Primary outcomes: Symptoms of generalized anxiety Depression
		Control (83.3%) Cognitive behavioral (75%)					BDI	
Poulsen et al. (2014) and Katznelson et al. (2020) (Denmark)	25.8 (4.9)	69/1	BN	Psychoanalytic psychotherapy (PPT)	70	72.3 (10.6)	Eating Disorder Examination interview (Fairburn and Cooper, 1993); Present State Examination (The SCAN Advisory Group, 1994); SCL-90-R; BDI-II, STAI	Primary outcome:cessation of binge eating and purging secondary outcomes: eating disorder psychopathology; general psychopathology therapeutic alliance
				CBT		22	Vanderbilt Therapeutic Alliance Scale (VTAS; Hartley and Strupp, 1983) Adult Attachment Interview (AAI; George et al., 1996)	Attachment relationships
							Reflective Function Scale (Fonagy et al., 2016)	
Rahmani et al. (2020) (Kurdistan)	23.07 (3.26)	22/19	Social anxiety disorder (SAD)	Intensive short-term dynamic psychotherapy (ISTDP)	41	10	DSM-5 criteria for SAD; Liebowitz Social Anxiety Scale (Liebowitz, 1987)	Primary outcomes: target symptom (fear and avoidance)
Stefini et al. (2017) (Germany)	18.7 (1.9)	81/0	BN	Psychodynamic therapy (PDT)	42	33.0 (25.3)	SCID-I and SCID-II for the DSM-IV Eating Disorder Examination Interview (EDE)	Primary Outcome: Remission from BN
				CBT	39	40.7 (22.2)	SCL-90-R EDE Questionnaire (EDE-Q)	Secondary outcomes: severity of BN symptoms; psychiatric comorbidities; overall severity of mental symptoms
Zipfel et al. (2014), Egger et al. (2016), Herzog et al. (2022) (Germany)	27.7	242/0	AN	FPT	80	NR	BMI; EDI; Structured Interview for Anorexia and Bulimia Nervosa for DSM-IV and ICD-10 (SIAB-EX, Fichter et al. 1991)	Primary outcome: Weight gain
				CBT	80			
				Optimized treatment as usual (TAU-O)	82	NR		Secondary outcomes: rate of recovery (combination of weight gain and eating disorder-specific psychopathology)
Walton et al. (2020) (New South Wales, Australia)	26.6 (7.8)	125/37	BPD	Dialectical behavior therapy (DBT)	162	112	Combined outcome of any episode of suicidal and non-suicidal self-injury (SASI)-Count (Linehan and Comtois, 1996; Linehan et al., 2011) BDI-II	Primary Outcomes: Number of suicidal attempts and non- suicidal self-injurious (NSSI) episodes Depression severity
				Conversational model (CM)		112	Borderline Personality Disorder Severity Index (BPDSI-IV; Arntz et al., 2003). Inventory of Interpersonal Problems (IIP; Horowitz et al., 1988); Dissociative Experiences Scale (DES; Bernstein and Putman, 1986); Sense of Self Inventory (SSI; Basten, 2008);	Secondary Outcomes: BPD Severity, Interpersonal problems, Dissociation, Sense of self, Mindfulness, Emotion regulation
							Kentucky Inventory of Mindfulness Skills (KIMS; Baer et al., 2004); The Difficulties in Emotion Regulation Scale (DERS; Gratz and Roemer, 2004)	
**Naturalistic studies**								
Baruch and Fearon (2002), Baruch (1995), Baruch et al. (1998) (United Kingdom)	19.7 (3.2)	102/49	Principal ICD-10 diagnosis (World Health Organization, 1992): mood disorder (*n* = 53%) Conduct disorder (11%) Neurotic disorder, stress-related, or somatoform disorder (20%) Personality disorder (8%)	Psychodynamic psychotherapy	151	66	Young adult self report form (YASR; Achenbach, 1997)	Primary outcome: internalising and externalising problems
Gerber et al., 2004 (United Kingdom)	22.8 (2.1)	17/8	Depression, anxiety, and personality disorders DSM-III-R diagnoses	Psychodynamic psychotherapy	11	6 months to 8 years long	BDI STAI-T SCID-II	Primary outcomes: anxiety and depression symptoms
				Psychoanalysis	14		Schedule for Affective Disorders and Schizophrenia (SADS; Endicott and Spitzer, 1987)	Secondary outcomes: DSM diagnoses Axis I and II
Harder et al. (2014) and Rosenbaum et al. (2012) (United Kingdom)	24	88/181	First-episode schizophrenia spectrum disorder	Supportive psychodynamic psychotherapy (SPP)	119	3 years	Operational criteria checklist for psychotic illness (OPCRIT; McGuffin et al., 1991) GAF-symptom, GAF-function	Primary outcome: Psychosocial functioning
			(SSD)	Standard Treatment (ST)	150		Strauss-Carpenter scale (Strauss and Carpenter, 1974, 1977) Positive and negative syndrome scale (PANSS; Kay et al., 1987)	Secondary outcome: target symptoms
Nemirovski Edlund and Carlberg (2016) (Sweden)	19.17 (2.45)	166/52	Mood disorder (*n* = 69) Anxiety disorder (*n* = 59) Other diagnoses (*n* = 69)	Psychodynamic psychotherapy	218	43 (50)	SCL-90 children’s global assessment scale (CGAS, Shaffer et al., 1983) GAF	Primary outcomes: general functioning and symptoms severity
Falkenström (2010) (Sweden)	Sample 1: 19.1 (2.9)	312/104	Mood (30%) and anxiety (24%) disorders	Psychodynamic	416	23 (19)	SCL-90; IIP; GAF	Primary outcomes: defense mechanisms and copying functioning
	Sample 2: 19 (1.8)	83/18	Personality disorder	Psychotherapy	101			
Kramer et al. (2010, 2014) (Switzerland)	24 (3.86)	26/6	Adjustment disorder with depressed mood personality disorder cluster B	Short-term dynamic psychotherapy (STDP)	32	40	Defense mechanism rating scales (Perry, 1990) and coping action patterns (Perry et al., 2005)	Primary outcomes: general functioning and symptoms severity
							SCL-90-R; BDI–II;	Secondary outcomes: depression therapeutic alliance affective meaning states
							Helping Alliance Questionnaire—II (HAq–II; Alexander and Luborsky, 1986)	
							Classification of affective meaning states (CAMS; Pascual-Leone and Greenberg, 2005)	
Philips et al. (2006), Lindgren et al. (2010), Werbart et al. (2017) Sweden (Young adult psychotherapy project)	22 (2.2)	98/36	Personality disorders; non diagnosed depressive mood, anxiety, problems in the relationship to parents, and low self-esteem	Individual psychoanalytic psychotherapy (IPP)	92	15 months	DSM-IV and ICD-10 personality questionnaire (DIP-Q; Ottosson et al., 1995) SCL-90; BSI; Self-rated health (SRH; Bjørner et al., 1996)	Primary outcomes: personality disorder
				Group psychoanalytic psychotherapy (GPP)	42		GAF; IIP; The structural analysis of social behavior intrex questionnaire (SASB; Benjamin, 1988) The differentiation-relatedness of self and object representations scale (DRS; Blatt and Auerbach, 2001) HAq-II	Secondary outcomes: target symptoms, psychosocial function
Riva Crugnola et al. (2020) (Italy)	23.29 (4.89)	96/28	N/A Self-referred University students	Brief psychodynamic counselling	124	4	SCL-90-R	Primary outcome: severity of psychopathological symptoms
							Attachment style questionnaire (ASQ; Feeney et al., 1994; Italian version: Fossati et al., 2003)	Secondary outcome: attachment style

AAI, adult attachment interview; AN, anorexia nervosa; APT, affect phobia therapy; ASAD, adult separation anxiety disorder; ASA-27, adult separation anxiety questionnaire; ASQ, attachment style questionnaire; BEP, brief empathic psychotherapy; BN, bulimia nervosa; BDI-II, beck depression inventory; BMI, body mass index; BSI, brief symptom inventory; BPD, borderline personality disorder; BPDSI-IV, borderline personality disorder severity index; CAMS, classification of affective meaning states; CGAS, children’s global assessment scale; CAT, cognitive- analytic therapy; CBT, cognitive behavioral therapy; CISSB, cornell interview for suicidal and self-harming behavior; CM, conversational model; C/NC, control/nutritional counseling only; COT, cognitive orientation treatment; CRTHI, Cornell revised treatment history inventory; DASS-21, depression, anxiety, and stress scale-21; DBT, dialectical behavior therapy; DERS, the difficulties in emotion regulation scale; DES, dissociative experiences scale; DIP-Q, DSM-IV and ICD-10 personality questionnaire; DRS, differentiation-relatedness of self and object representations scale; DSM, diagnostic and statistical manual of mental disorders; EAT 26, eating attitudes test; EPDS, Edinburgh postnatal depression scale; FPP, focal psychodynamic psychotherapy; FT, family therapy; GAF, global assessment of functioning; GSI, global severity index; GPP, group psychoanalytic psychotherapy; HAq–II, helping alliance questionnaire—II; HRSA, Hamilton rating scale for anxiety; KIMS, Kentucky inventory of mindfulness; IIP, inventory of interpersonal problems; IPP, individual psychoanalytic psychotherapy; ISTDP, intensive short-term dynamic psychotherapy; LRT, low contact, `routine’ treatment; MDD, major depressive disorder; NSSI, non- suicidal self-injurious; OPCRIT, operational criteria checklist for psychotic illness; PANSS, positive and negative syndrome scale; PPT, psychoanalytic psychotherapy; SAD, social anxiety disorder; SADS, schedule for affective disorders and schizophrenia; SASB, structural analysis of social behavior intrex questionnaire; SASI, suicidal and non-suicidal self-injury; SCID, structured clinical interview for DSM; SCI-SAS, structured clinical interview for social anxiety symptoms; SCL-90, symptom checklist-90; SET, supportive-expressive therapy; SIAB-EX, structured interview for anorexia and bulimia nervosa; SPIN, social phobia inventory; SPP, supportive psychodynamic psychotherapy; SPT, self- psychological treatment; SSD, schizophrenia spectrum disorder; SSI, sense of self inventory; ST, standard treatment; STAI, state–trait anxiety inventory; STDP, short-term dynamic psychotherapy; TAU-O, optimized treatment as usual; TFP, transference-focused psychotherapy; VTAS, Vanderbilt therapeutic alliance scale; WCST, Wisconsin card sorting test; YASR, young adult self report form.

The review identified 15 studies evaluating psychodynamic treatment for participants with an emotional disorder, including depression (*n* = 8) and anxiety (*n* = 7). A total of six studies focused on individuals with eating disorders and a further six on individuals with personality disorders. One study included participants with psychosis.

With the exception of one study on group psychoanalytic psychotherapy, all studies included individual psychodynamic therapy. The majority of studies (*n* = 14) were conducted in European countries, with the remainder being conducted in Asia (*n* = 6), Australia (*n* = 1) and Canada (*n* = 1).

There was some consistency in the therapy aims. About half of the studies (*n* = 13) focused on symptom reduction as the primary target. Six studies included assessment of psychosocial functioning. The interventions ranged from once or twice a week Psychoanalytic Psychotherapy to Intensive short-term dynamic psychotherapy, Self- psychological treatment, Supportive Psychoanalytic Psychotherapy, Focal psychoanalytic psychotherapy, manualized treatments like Transference-focused psychotherapy, and Dynamic Interpersonal Therapy, Supportive-Expressive Therapy, Conversational model (psychoanalytic treatment for BPD), Brief Empathic Psychotherapy, Integrative therapy. Only one study included five times a week Psychoanalysis (Gerber et al., 2004). The length of the treatments varied in terms of duration spanning from a minimum of four sessions up to 8 years. A total of 15 studies included a follow-up period, ranging between 2 months to 5 years after therapy termination.

### 3.3 Narrative synthesis of outcomes measures and findings from RCTs and naturalistic studies

We grouped the outcome measures into five categories across the included studies, including clinical symptoms, recovery/relapse, psychosocial functioning, personality structure and interpersonal relationships. A total of 9 studies (both RCT and naturalistic), reported the Global Severity Index scale of the Symptom Checklist 90 Revised (SCL-90-R; Derogatis and Unger, 2010) as the main clinical outcome measure for psychiatric symptoms, and showed a significant decrease in psychopathological symptoms from baseline to end of psychodynamic treatment (Nemirovski Edlund and Carlberg, 2016; Falkenström, 2010; Riva Crugnola et al., 2020; Stefini et al., 2017). A naturalistic study, the Young Adult Psychotherapy Project (Philips et al., 2006), showed that improvements on the overall psychological functioning measured using the SCL-90 and the Global Assessment of Functioning Scale (GAF; American Psychiatric Association, 2013) were maintained also at follow-up 1.5 and 3 years after termination (Philips et al., 2006; Lindgren et al., 2010; Werbart et al., 2012, 2017). Similarly, a significant decrease in psychotic symptoms was maintained for 5 years after psychodynamic treatment in young people experiencing a first psychotic episode (Harder et al., 2014).

Several studies focused on emotional disorders and affective symptoms, such as depression (*n* = 6), anxiety (*n* = 4) and internalizing/externalizing problems (*n* = 1). Both self-report (using the Beck Depression Inventory, BDI; Beck et al., 1996) and interviewer-rated symptoms of depression (i.e., using the Structured Clinical Interview for DSM; American Psychiatric Association, 2013) were significantly lower after psychodynamic psychotherapy and at follow-up compared with the wait-list control group (Ajilchi et al., 2016; Walton et al., 2020). However, a study on post-partum depression in young mothers showed that the benefit of treatment on depression was no longer significant by 9 months post-partum (Cooper et al., 2003).

One study that evaluated the outcomes of psychoanalysis for young adults showed that improvement in levels of depression and anxiety and number of Axis I diagnosis scales achieved by the end of treatment termination was maintained 18 months after termination (Gerber et al., 2004). Young people who received community-based psychodynamic psychotherapy reported a decrease in internalizing and externalizing problems after 1 year in treatment (Baruch and Fearon, 2002).

Studies of short-term psychodynamic treatment also showed significant differences in the generalized anxiety and depression symptom scores between pre-treatment, post-treatment, and follow-up stages in the experimental group compared to the control group (Mowlaie et al., 2018; Orvati Aziz et al., 2020), with a significant reduction of fear and avoidance symptom scores in the young adults with social anxiety and social phobia in psychodynamic treatment compared to those in the control group (Moghadam et al., 2015; Rahmani et al., 2020).

A few studies focused on secondary outcomes, including dropouts from treatment (*N* = 1), suicide attempts (*N* = 2), self-harming (*N* = 2), and eating disorder psychopathology, including recovery (*N* = 1), inpatient admission/relapse (*N* = 1), vomiting or binging (*N* = 3) and weight gain (*N* = 1). A significant decrease over time in suicidal and/or self-harming episodes and psychiatric inpatient admissions was observed in two RCTs focusing on psychodynamic psychotherapy for young people with a diagnosis of borderline personality disorder (Doering et al., 2010; Walton et al., 2020).

In terms of eating disorders, there was a significant weight gain in the Psychodynamic Psychotherapy group after 1 year of treatment compared to the routine treatment group (Dare et al., 2001) and higher remission rates in young adults in psychodynamic treatment group compared to CBT (Stefini et al., 2017).

When compared to CBT, psychoanalytic psychotherapy was shown to be effective in reducing purging and binge eating (Garner et al., 1993; Stefini et al., 2017) and remission from bulimia occurred for 15% of participants in the psychoanalytic psychotherapy group at 2 years follow-up (Katznelson et al., 2020; Poulsen et al., 2014). However, the improvements in eating disorder symptoms and general psychopathology occurred more rapidly in the patients receiving the CBT intervention (Garner et al., 1993; Katznelson et al., 2020; Poulsen et al., 2014).

A minority of studies assessed psychosocial functioning (*n* = 6), including a global measure of functioning and social behavior. When compared to other talking therapies, Transference Focused Psychotherapy showed efficacy in the domain of social functioning in people with borderline personality (Doering et al., 2010). The treatment effect of psychodynamic psychotherapy on social functioning increased over 2 years after treatment but became non-significant at 5-year follow-up (Harder et al., 2014; Rosenbaum et al., 2012).

One study also assessed executive functioning, with the psychodynamic group significantly improving on executive functioning at post-treatment compared to young adults who were in the wait-list group (Ajilchi et al., 2016).

Another category was represented by outcomes related to the assessment of personality structure (Personality Disorder diagnosis *n* = 4, dissociation *n* = 1, sense of self *n* = 1, mindfulness *n* = 1, emotion regulation *n* = 1, defense mechanisms *n* = 1, affective meaning states *n* = 1). BPD severity, personality organization, dissociation or sense of self showed significant improvement over time at the end of psychodynamic treatment (Doering et al., 2010; Walton et al., 2020). Short term psychodynamic therapy had an effect on overall defensive functioning, but no significant change for overall coping functioning (Kramer et al., 2010, 2014).

A handful of studies focused on interpersonal aspects, including constructs such as therapeutic alliance (*n* = 3), attachment style and reflective function (*n* = 2), interpersonal problems (*n* = 2), experience of the therapy (*n* = 1), self and object representations (*n* = 1). The Young Adult Psychotherapy Project found positive changes with regard to reflective function, interpersonal problems and self and object representations (Katznelson et al., 2020; Lindgren et al., 2010; Philips et al., 2006; Werbart et al., 2017). However, another study found no moderation effect of attachment styles on the effectiveness of the psychodynamic intervention (Riva Crugnola et al., 2020).

Overall, the majority of the reviewed studies showed that psychoanalytic psychotherapy had a significant positive effect on young adults involved in the treatment, including symptoms reduction, remission, psychosocial and interpersonal functioning, and improvement on personality outcomes.

#### 3.3.1 Quality assessment

**Figure 2:**
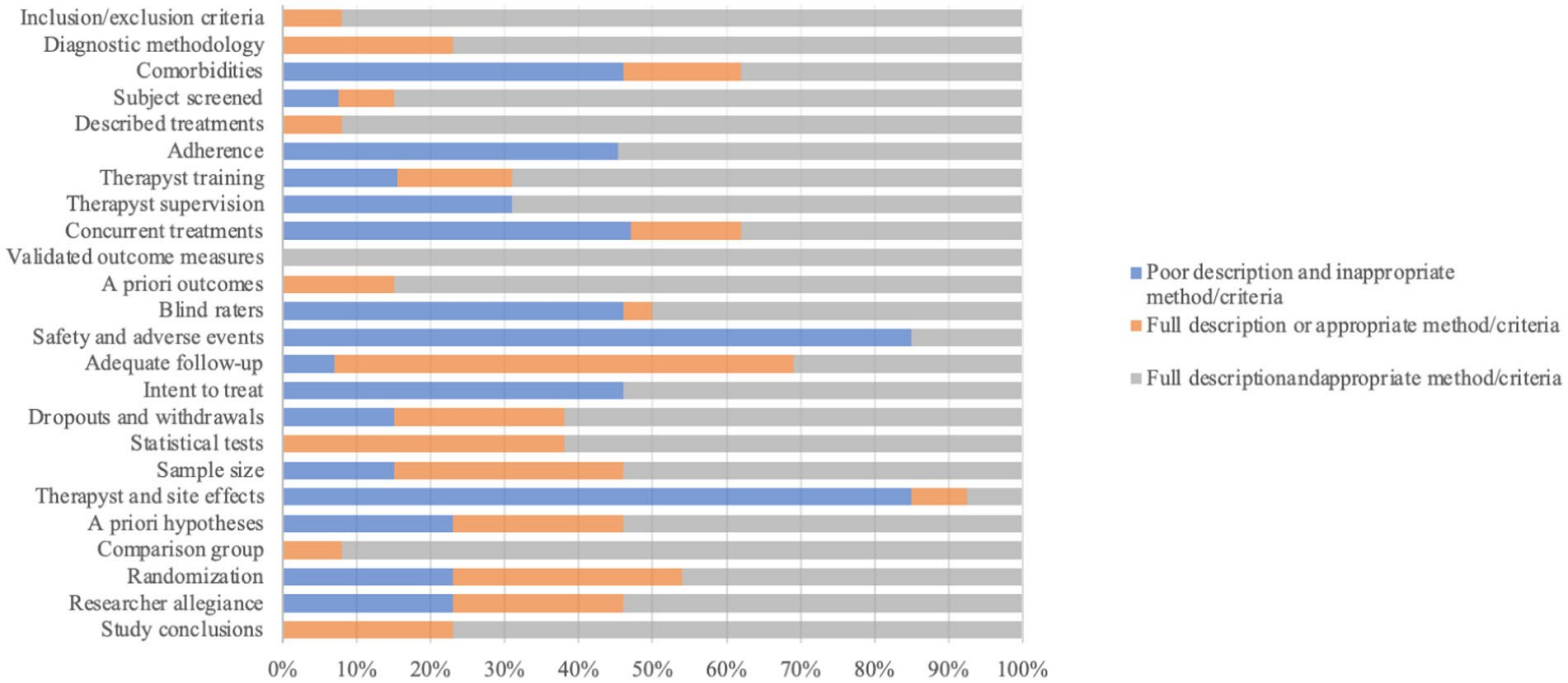
Percentage of randomized controlled trials (RCTs) for psychodynamic psychotherapy (*N* = 14) by item score on the RCT-psychotherapy quality rating scale. Items are rated from 0 to 2.

Figure 2 outlines the overall quality score for all 14 trials included within the review, on each item assessed using the RCT-PQRS.

Quality assessment scores were variable across RCTs, with an average total RCT-PQRS score of 33.4 (SD: 9.8, range 15–42). Eight studies rated as ‘good’ or ‘exceptionally good’ quality, 4 rated as ‘moderately good’ or ‘average quality’, and 2 as ‘moderately poor’ quality. More than half of the studies scored as ‘good’ (rating of 2) on items pertaining to description of inclusions and exclusion criteria, description of participants’ characteristics, treatment adherence and therapist supervision, use of validated outcome measures, comparison group from similar population, intention to treat analyses and justified conclusions.

For 12 studies, quality scores were poor (rating of 0) for reporting safety and adverse events and consideration of site effects. Out of the 14 RCTs with published results, 5 had fewer than 25 people in the treatment arm. Finally, four of the RCTs used treatment as usual as the control arm and a similar number included both a control arm and a CBT treatment arm.

The overall quality was ‘fair’ for the majority of the naturalistic studies (*n* = 8), with one study classified as ‘good’ quality (Harder et al., 2014). However, in more than half of the studies, attrition bias, with most studies reporting >20% loss to follow-up at the end-point, and masking of outcome assessors were cause for concern.

#### 3.3.2 Overall effects of psychoanalytic psychotherapy

The second aim of the study was to carry out a meta-analysis. It was not possible to carry out a meta-analysis of the naturalistic studies, due to the majority of the studies not including a control or comparison group. To that effect we firstly compared psychoanalytic treatments with other psychological or pharmacological interventions for the 14 RCTs included.

**Figure 3:**
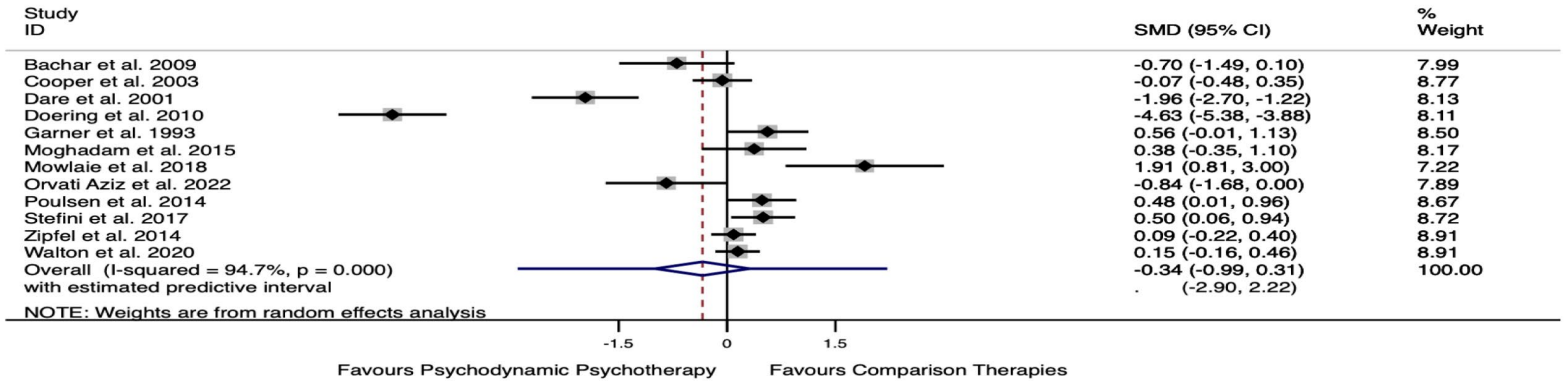
Forest plot of the effect of psychodynamic psychotherapy compared to other treatments for the primary outcomes.

Figure 3 shows the forest plot of the effect of psychoanalytic therapy compared to other treatments for the primary outcome. The figure shows the effect estimates from the single RCT studies and the pooled result.

The standardized mean difference between psychoanalytic psychotherapy and other treatments was of 0.3, which suggests a small effect of psychoanalytic psychotherapy compared to other psychotherapies or pharmacotherapies at post-treatment, however this did not reach statistical significance [SMD of −0.341 (95% CI: −0.991;–0.309), *p* = 0.304].

Heterogeneity was high for the primary outcome post-therapy (*I*^2^ = 95%), as a consequence of the clinical and methodological diversity among the studies. Studies in which the 95% confidence interval was outside the 95% confidence interval of the pooled studies were considered outliers. In our meta-analysis, one outlier was identified within the clinical population subgroup (Doering et al., 2010). The removal of the outlier did not change the results in terms of outcome improvement at the end of treatment [SMD of −0.04 (95% CI: −0.34;0.41), *p* = 0.843]. Heterogeneity was reduced but was still significant [*Q*(9) = 58.3, *p* < 0.001, *I*^2^ = 83%].

**Figure 4:**
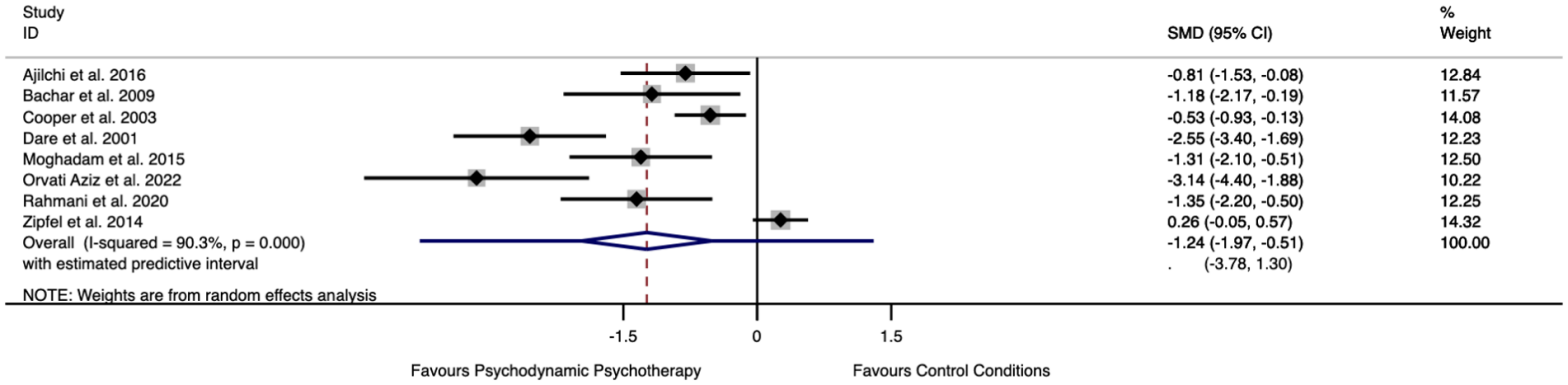
Forest plot of the effect of psychodynamic psychotherapy compared to control groups for the primary outcomes.

Only a total of eight RCTs provided data on the control group (e.g., patients on waiting list or receiving treatment as usual). The meta-analysis yielded a standardized mean difference (SMD) of −1.24 (95% CI: −1.97;–0.51, *p* < 0.001), which suggests that young adults who received a psychoanalytic intervention showed a significant improvement on outcomes at the end of treatment of more than 1 standard deviation above those in the control group (Figure 4). There was also significant heterogeneity between this subgroup of studies [*Q*(6) = 72.46, *p* < 0.001, *I*^2^ = 90.3%].

We conducted sensitivity analyses to test for the effect of psychoanalytic psychotherapy at follow-up. Only four RCTs measured the effect of psychodynamic therapy compared to control conditions after the end of treatment. The meta-analysis (Supplementary Figures S1) shows that the effect of psychoanalytic therapy was attenuated but remained statistically significant at follow-up [SMD = −0.75 (95% CI: −1.53;0.03, *p* < 0.001)].

Secondary outcome analyses show similar effects of psychoanalytic psychotherapy compared to other therapies and control groups at post-treatment (Supplementary Figures S2, S3).

The main results therefore did not show significant differences between psychoanalytic treatments and other psychological or pharmacological treatments on primary outcomes. However, there was a statistically significant effect of psychoanalytic psychotherapy for young adults on both primary and secondary outcomes at the end of treatment and follow-up, compared to young adults who were in the control group and did not receive psychoanalytic treatment.

#### 3.3.3 Moderator analyses and publication bias

We examined for moderating effect of year of publication, quality score, age, sex, outcome measure, recruitment method, intent-to-treat, diagnosis, patient-per therapist ratio, and average sample size per group. The meta-regression analyses showed no impact on the effect sizes (Supplementary Table S1). Egger’s regression test did not show funnel plot asymmetry (intercept = 0.92, 95% CI = -1.0.8 to 2.91, *p* = 0.326) for the studies comparing psychodynamic psychotherapy with other treatments. However, there was evidence for publication bias in the meta-analysis comparing psychodynamic psychotherapy with control conditions (intercept = 1.06, 95% CI = 0.10 to 2.02, *p* = 0.036).

## 4 Discussion

To our knowledge, this is the first meta-analysis in the field to systematically examine the efficacy of psychoanalytic psychotherapy specifically for the young adult population, which is an under-researched area. Overall, our review highlights a range of methodological limitations of published studies, and the need to conduct further research focusing on this developmental phase to better investigate the efficacy of psychodynamic psychotherapy for young people. It was a surprising finding that despite the increase of empirical research in this field, only 14 studies included a robust RCTs design, of which only 8 were assessed a good or exceptional quality.

Our review highlighted that when compared to a range of other treatments, including CBT, psychodynamic psychotherapy showed no significant difference in efficacy. These findings are in line with previous review and meta-analyses that focused on children or adult populations (Nemirovski Edlund and Carlberg, 2016; Midgley et al., 2021; Steinert et al., 2017). The results showed an effect of psychoanalytic therapy in young adults when compared to those young adults who were randomized into a control group (*g* = −1.24 [95% CI: −1.97;–0.51], *p* < 0.001). These effects were maintained at follow-up [*g* = −0.75 (95% CI: −1.53;0.03), *p* < 0.001]. Our meta-analysis therefore added to the existing evidence by showing that psychoanalytic treatments are also effective for young people transitioning from adolescence to adulthood.

Most studies examined in the review focused on symptom reduction as the primary outcome of their intervention. However, improvements were also demonstrated across a wide range of outcome indicators, including general psychopathological symptoms measured with the SCL-90 and Global Assessment of Functioning Scale (GAF), personality and social functioning. Despite the majority of studies included in this review used symptom-oriented outcome measures, the overall aim of psychodynamic therapy goes well beyond symptomatic remission. Shedler (2010) has highlighted the discrepancy between the goals of psychodynamic therapy and the measures typically used in outcome studies, who might not capture the extent of benefits of psychodynamic interventions in terms of the person’s inner resources and capacity to live a more fulfilling life.

Moreover, our narrative synthesis highlighted similar findings derived from naturalistic studies. The advantage of such studies is that they provide a realistic picture of how psychodynamic therapies impact the lives of young adults accessing mental health treatments (Ross and Naylor, 2017). Compared to randomized controlled studies, who have narrower inclusion criteria and include a more omogeneous population, naturalistic studies investigate young people treated in everyday clinical settings and allow to examine the effect of psychodynamic psychotherapy on young adults within a clinical practice. Their findings are thus are closer to clinical reality and more likely to be generalizable (Weisz et al., 1992).

## 5 Limitations

The present study has several limitations. We could include only a small number of studies in the review, and a limited number of clinical trials in our meta-analysis, which limit the generalisability of findings and highlights the neeed to produce more high quality RCTs. Furthermore, most of the studies included focused on short term interventions, while in clinical practice, psychodynamic interventions are often long term and open ended.

Another limitation of the findings is the high heterogeneity identified in the published RCTs. This suggests that the effects differed considerably across studies. There are several possibilities that could account for that, including that the psychoanalytic interventions, treatment durations, psychopathological presentations as well as the control treatments of the studies included were diverse and varied greatly. Some studies, for example, offered brief interventions over 4 weeks whereas some offered more intensive interventions over 2 years. This raises important questions as per the generalisability and clinical value of the findings.

A way to reduce the heterogeneity would have been to conduct subgroup analyses, however this was not possible due to the small sample of studies available and again points to the importance of future researchers to conduct further high-quality studies. Thus, overall, our results can only be seen in light of these important limitations and as such should be interpreted with caution.

Lastly, in our meta-analyses, we could not control for researchers’ allegiance, which has been repeatedly shown to bias results in psychotherapy research (Munder et al., 2011, 2013). In most of the studies, outcome assessors were not blind to treatments. Therefore, another limitation is represented by the risk of bias of the studies included, a topic that that needs to be addressed in future research.

### 5.1 Clinical and research implications

Around 75% of mental health disorders have their onset in young adulthood (Auerbach et al., 2018; Kessler et al., 2005). Yet, only a limited amount of evidence as per effective treatment options for these individuals is available. Our meta-analytic study of 14 RCTs has shown that psychoanalytic psychotherapy might be an effective treatment option alongside other psychotherapeutic approaches. It certainly is more effective than not receiving treatment at all (Hedges’*g* − 1.24 [95% CI: −1.97;–0.51], *p* < 0.001).

Many young adults who need support from mental health services are ‘lost’ to the system when they transition into adulthood (Mclaren et al., 2013; McNicholas et al., 2015; Singh et al., 2010). Those affected are often the most vulnerable and disadvantaged; getting lost during this transition increases the likelihood to be unemployed and out of education or training; it can also impact negatively on their physical health (Trotta et al., 2019). By showing that psychoanalytic therapies are effective in the treatment of the young adults, the findings from our review highlight the need to offer a range of treatment options, tailored to the young person’s needs.

A distinctive feature of psychodynamic interventions compared to other talking therapies is the focus on interpersonal relationships, the identification of recurring patterns in people’s functioning and in their feelings (Blagys and Hilsenroth, 2000; Luborsky et al., 1990). In understanding attitudes and feelings in the present, psychoanalytic approaches emphasize the importance of a developmental perspective as well as the role of insight into the past. There is a growing consensus on the importance of focusing on a young person’s internal world, on the unconscious factors that orient the perception of external reality and how these, in turn, might lead to the development and maintenance of pathological defenses. As such, psychoanalytic therapy employs a different mechanism of change than other therapies and therefore might be an important treatment options for some young people as this approach addresses specific aspects that other therapeutic modalities might not focus on (Blagys and Hilsenroth, 2000).

Taken together, these findings offer some initial evidence for the effectiveness of psychoanalytic treatment for young adults and highlight the need to conduct research to understand the efficacy of different treatment options for young adults and whether the benefits persist also in later follow-up. Therefore, this further highlights the importance of more robust and high quality studies focusing on young adulthood that aim to prevent and treat the progression of mental health and interpersonal difficulties later in life.

## 6 Conclusion

By showing some effect on the mental health and psychosocial functioning of young people, the findings of this systematic review have theoretical and clinical implications for the use of psychoanalytic psychotherapy to support the complex transition of young people into adulthood. Future research on psychoanalytic psychotherapy outcomes is warranted to identify the most effective psychoanalytic interventions for young adults and to tailor them to this specific population’s developmental needs. Future review on efficacy of psychodynamic treatments in adults including studies with langer sample size are recommended.
