# Supplementary material for: The efficacy of psychodynamic psychotherapy for young adults: a systematic review and meta-analysis
Source: Front Psychol. 2024 Sep 4;15:1366032. doi: 10.3389/fpsyg.2024.1366032 (PMC11408295; doi:10.3389/fpsyg.2024.1366032)
Supplement: Supplementary file 1 [file Table_1.DOCX]

**Supplementary Material**

**1. Main search strategy**

((((((psychodynamic* or psycho-dynamic* or dynamic* or psychoanalytic* or interpretive or expressive or STPP or LTPP or ISTDP)) AND (therapy or therapies or psychotherap* or treatment* or counselling)) AND (young or adult* or youth*)) AND (study or studies or trial*)) AND (outcome* or result or results or effect* or change*) AND (psychiat* or mental* or psychol* or diagnosis))

i) using the Pubmed, PsychINFO, Central, Embase classic and Embase, Epub Ahead of Print, In-Process & Other Non-Indexed Citations, Ovid MEDLINE(R) Daily and Ovid MEDLINE(R) databases.

Also: we conducted a search using the Cochrane Central Register of Controlled Trials, a registry of randomized controlled trials that has been developed through systematic search- es of MEDLINE, Embase, CINAHL, LILACS, the “gray literature” of unpublished results, and hand searches

ii) Manual searches in the following publications:

Abbass AA, Hancock JT, Henderson J, Kisely S. Short-term psychodynamic psychotherapies for common mental disorders. Cochrane Database Syst Rev. 2006:CD004687.

Barber JP, Muran JC, McCarthy KS, Keefe JR. Research on psychodynamic therapies. In: Lambert MJ, ed. Bergin and Garfield's Handbook of Psychotherapy and Behavior Change (6th ed). NY: Wiley;2013:443-494.

Driessen E, Cuipers P, deMaat SCM, Abbass A, deJonghe F, Dekker JJM. The effiacy of short-term psychodynamic psychotherapy for depression. A meta-analysis. Clin Psychol Rev. 2010;30:25- 36.

Driessen E, Hegelmaier LM, Abbass AA, et al. The efficacy of short-term psychodynamic psychotherapy for depression: A meta-analysis update. Clin Psychol Rev. 2015;42:1-15.

Leichsenring F, Leweke F, Klein S, Steinert C. The Empirical Status of Psychodynamic Psychotherapy - An Update: Bambi's Alive and Kicking. Psychother Psychosom. 2015;84:129-148.

Leichsenring F, Luyten P, Hilsenroth MJ, et al. Psychodynamic Therapy Meets Evidence-Based Medicine: A Systematic Review Using Updated Criteria. Lancet Psychiatry. 2015;2:648-660.

Keefe JR, McCarthy KS, Dinger U, Zilcha-Mano S, Barber JP. A meta-analytic review of psychodynamic therapies for anxiety disorders. Clin Psychol Rev. 2014;34:309-323.

plus: search of reference lists of included studies

plus: search in published and regularly updated list (“Lilliengren-List”) of all previously identified

RCTs on PDT (http://w3.psychology.su.se/staff/peli/RCTs_of_PDT.pdf)

**Supplementary Table 1**. Meta-regression for meta-analysis on the effect of psychodynamic psychotherapy compared to other treatments for the primary outcomes.

| **Moderators** | **B** | **SE** | **95% CI** | ***p*** |
| --- | --- | --- | --- | --- |
| Publication year | 0.036 | 0.055 | -0.09; 0.16 | 0.527 |
| Quality score | -0.043 | 0.049 | -0.15; 0.65 | 0.398 |
| Age | -0.284 | 0.162 | -0.65; 0.08 | 0.112 |
| Female % | -0.002 | 0.007 | -0.02; 0.13 | 0.782 |
| Outcome measures | -1.448 | 1.252 | -4.24; 1.34 | 0.274 |
| Recruitment method | -1.643 | 1.019 | -3.91; 0.63 | 0.138 |
| Diagnosis | -0.208 | 0.313 | -0.91; 0.50 | 0.523 |
| Intent-to-treat | -1.254 | 0.914 | -3.29; 0.78 | 0.200 |
| Patient-per therapist ratio | -0.056 | 0.114 | -0.22; 0.34 | 0.643 |
| Average sample size | 0.002 | 0.043 | -0.18; 0.19 | 0.962 |

Note: B= Standardised beta coefficient; SE= Standard Error; CI= Confidence Interval; *p*= *p* value.

**Figure S1.** *Forest plot of the effect of psychodynamic psychotherapy compared to control groups at follow-up.*

**Figure S2.** Forest plot of the effect of psychodynamic psychotherapy compared to other treatments for the secondary outcomes.

**Figure S3.** Forest plot of the effect of psychodynamic psychotherapy compared to control groups for the secondary outcomes.
